# Aortic Valve Replacement

**Published:** 1969-10

**Authors:** R. G. Charles, C. M. Morgans


### AORTIC VALVE REPLACEMENT: A FOLLOW-UP

**R. G. Charles and C. M. Morgans**

Marked stenosis or incompetence of the aortic valve invariably causes progressive disability and early death. They are best treated by surgery, and since July 1964 the treatment of choice in Bristol has been replacement with the Starr-Edwards ball valve prosthesis. Originally constructed of a silicone rubber ball contained in a Vitallium-like alloy cage, the ball has shown signs of wearing out after some years and the newer model contains a steel ball which is thought also to reduce the risk of thrombus formation on the valve.

This report reviews patients who have left hospital following aortic valve replacement with regard to their quality of life, clinical progress and complications, and causes of death. Patients who had more than one valve replaced are excluded.

### PATIENTS AND METHODS

Between July 1964 and January 1969, 60 patients have left hospital following aortic valve replacement with the Starr-Edwards prosthesis.

All patients were fully investigated before operation. Catheter studies showed that all were in left ventricular failure and when these results were considered with clinical and electrocardiographic findings it was estimated that all patients had reached a life expectancy of two years or less. In this context it is important to note that 7 patients have died during the three-month waiting period for surgery.

Details of the 46 surviving patients are summarized below. Follow-up periods ranged from 4 to 57 months.

**Long Term Survivors**

| | Number of patients |
|---|---|
| Total ... ... ... | 46 |
| Men ... ... ... | 36 |
| Women ... ... ... | 11 |
| Lesion | |
| Aortic stenosis ... | 31 |
| Aortic incompetence ... | 15 |

Operation was performed for both aortic stenosis and aortic incompetence. The length of history ranged from two months to twelve years but it is important that in 35 patients the first symptom appeared less than five years before operation. The age range was 21-61 although most patients fell into the upper age groups.

| Age range | | | | | |
|---|---|---|---|---|---|
| No. of patients ... | 4 | 8 | 12 | 18 | 4 |

### RESULTS

Follow-up has shown that with the exception of four patients who will be mentioned later, all are well and express great satisfaction with the result of the operation.

Of paramount importance to the patient is that symptomatic relief has been striking and that the quality of life has improved. The Table below shows the common presenting symptoms, and indicates that effort syncope and the manifestations of left ventricular failure have been abolished. When angina remains it is reduced in severity. All patients with congestive cardiac failure were relieved by operation, two patients having subsequently developed it in association with reflux around the prosthesis.

**Effect of Aortic Valve Replacement**

| | Pre-op. | Post-op. |
|---|---|---|
| PND/LVF ... ... ... | 19 | 0 |
| Effort syncope ... ... ... | 10 | 0 |
| Angina ... ... ... ... | 29 | 3 |
| Congestive cardiac failure ... | 16 | 2 |

Exercise tolerance was graded according to shortness of breath (S.O.B.) in the 45 patients personally available for follow-up.

**Exercise tolerance after operation in 45 patients**

| | |
|---|---|
| No change ... ... | 7 |
| Improved ... ... | 37 (+ 1 severe angina) |
| Deteriorated ... ... | 0 |

| Grade | Number of patients Pre-op. | Post-op. |
|---|---|---|
| 0 Normal. No S.O.B. ... ... ... ... ... | 2 | 22 |
| 1 S.O.B. on normal exertion ... ... ... ... | 5 | 16 |
| 2 S.O.B. on < normal exertion; can climb stairs ... | 17 | 7 |
| 3 S.O.B. on flat—unable to climb stairs ... ... | 20 | 0 |
| 4 S.O.B. at rest (+ 1 severe angina) ... ... ... | 0 | 0 |

No patient was dyspnoeic at rest before operation, but whereas the majority were significantly restricted in their activity, the majority now undertake normal activity with either minimal shortness of breath or none at all. Angina prevented one man from taking exercise before operation; he is now in Grade 1 with only occasional angina.

Also of importance is the patient's capacity to earn a living or cope with housework. Of 19 patients who were off work, including housework, for periods up to seven years before operation, 14 have returned to full or part-time work. Ten patients are currently off work, as a result of recent operation (5), cerebral embolus (1), bacterial endocarditis (1), and social factors (3).

On the whole, a reduction in the number of drugs taken by the patient has not been achieved and many are taking more. This is largely accounted for by the fact that the majority are anticoagulated and in many cases digoxin and diuretics have been continued as an insurance policy.

In a number of patients there has been a reduction in heart size on X-ray and an improvement in the electrocardiogram may also occur.

### COMPLICATIONS

Clearly, aortic valve replacement has much to offer, and while the clinical progress of many patients has been uninterrupted, delayed complications have occurred.

**Complications in 46 Long Term Survivors**

| | Number of patients |
|---|---|
| Cerebral embolus ... | 12 |
| Pulmonary embolus ... | 1 |
| Bacterial endocarditis ... | 1 |
| A-V block ... ... | 1 |
| Anaemia ... ... ... | 17 |
| Aortic reflux ... ... | 21 |

The problem of systemic embolus after aortic valve replacement is linked closely with that of long term anticoagulation. Initially, patients were not routinely anticoagulated in Bristol. During this time 11 patients suffered episodes which were attributed to cerebral embolus but only one of these, a woman of 52, has suffered permanent sequelae and is now confused and dysarthric. Following the death of a patient from cerebral embolus in February 1966 the policy was changed and all patients are anticoagulated from the early post-operative period. Only one patient suffered a possible cerebral embolism while on anticoagulant therapy—this amounted to a 15-minute episode of dysarthria with no evidence of paresis. However, the problem of long term anticoagulation remains widely debated and the results of a long term trial must be awaited.

Pulmonary embolus was suggested in one patient by the onset of severe right chest pain with mild breathlessness, calf pain, and pyrexia. Chest X-ray was normal and there was no absolute proof.

One man developed bacterial endocarditis four weeks after operation, and blood cultures grew micrococci. There were also signs of aortic incompetence and heart failure. The Starr-Edwards prosthesis in this patient has been replaced by a homograft at the National Heart Hospital.

Only one patient developed persistent high-degree atrio-ventricular block after surgery though two others have left bundle-branch block. This complication may be caused by damage to the conducting system as the diseased valve is removed, or by sutures.

In 17 patients the haemoglobin concentration was lower than the standards of 13.5 gm/100 ml. for men and 12 gm/100 ml. for women. In 12 patients red cell fragmentation accompanied the low haemoglobin suggesting haemolysis. Eight of these patients had aortic reflux but there was no constant relationship between the degree of anaemia and the presence or degree of aortic reflux in this series. Brodeur and his colleagues (1965) have demonstrated a decreased red cell survival time in patients with aortic valve prostheses, but anaemia only appeared when the presence of aortic reflux added extra turbulence.

An early diastolic murmur was detected in 21 patients indicating reflux associated with the prosthesis. The murmurs were graded out of 4, grade 4 being accompanied by a thrill, and the distribution of patients is shown below.

**Aortic Reflux following Replacement**

| Grade | Number of patients |
|---|---|
| 1 | 7 |
| 2 | 9 |
| 3 | 5 |
| 4 | 0 |

Only two of these patients are unwell as a result of the reflux and they have congestive cardiac failure. In both these patients the aortic valve was grossly calcified and in one case this involved the wall of the aorta. This greatly increases the problems of resection and fixation of the prosthesis. In one man who came to surgery for re-insertion of the prosthesis, half the sutures were found to have cut through the aortic valve ring and it is probable that the other cases of aortic incompetence are caused by detachment of some of the anchoring sutures. Scannell and Austen (1966) found this an important cause in seven of their patients with significant regurgitation.

Long term anticoagulation has been attended by complications in eight patients. A total of 39 patients are on long term therapy. The number of patients showing individual complications is shown below.

**Complications of Anti-coagulation**

| | Number of patients |
|---|---|
| Alimentary bleeding ... | 4 |
| Haemoptysis ... ... | 2 |
| Haematuria ... ... | 2 |
| Epistaxis ... ... | 2 |
| Haemarthrosis ... | 1 |
| Haematoma ... ... | 1 |

Two patients with epistaxis required hospital treatment but all responded to adjustment of the dosage and have remained on anticoagulant therapy.

DEATHS AFTER DISCHARGE

Fourteen patients leaving hospital after aortic valve replacement have died. The causes of death and length of survival are shown below.

**14 Death after Discharge—Causes**

| | No. of patients | Survival (months) |
|---|---|---|
| Unrelieved L.V.F. ... | 1 | 3 |
| Myocardial infarction ... | 2 | 22, 38 |
| Clotted prosthesis ... | 1 | 24 |
| Valve detachment ... | 4 | 26, 7, 7, 4 (*1 case) |
| Q fever endocarditis ... | 1 | 16 |
| Bronchopneumonia ... | 1 | 23 |
| Cerebral embolus ... | 1 | 3½* |
| Cardiac failure ... | 1 | 15* |
| Unknown ... ... | 2 | 2½, 22 |

(*clinical diagnosis only)

One man of 43 died from unrelieved left ventricular failure after operation for aortic incompetence and at post-mortem the left ventricle was found to be massively dilated. No other cause was found and it must be assumed that the failing myocardium had reached the stage at which recovery was impossible even by correction of the underlying valvular defect.

Two men aged 55 and 58 died following myocardial infarction. The underlying cause is difficult to identify with certainty. It is difficult to establish whether coronary embolism occurred. Furthermore, men of this age commonly sustain myocardial infarction quite unassociated with any cardiac surgery. One man presented clinically with myocardial infarction but was shown at post-mortem to have massive thrombus formation around the prosthesis which also occluded the left coronary ostium.

Valve detachment was diagnosed at post-mortem in three patients, and clinically in one man who developed severe congestive failure and a diastolic murmur after valve replacement.

Q fever endocarditis was confirmed by histological and serological evidence in one case which is to be fully reported elsewhere. One man had a laryngectomy for a suspected malignant tracheal stenosis but succumbed with bronchopneumonia after operation. Cerebral embolus killed one woman aged 47 and this led to the introduction of routine anticoagulation as described. At post-mortem a rim of material composed of fibrin and platelets surrounded the base of the prosthesis, and the same material was demonstrated occluding the lumen of the middle cerebral artery. In the remaining three patients no diagnostic data were available.

### CONCLUSION

Aortic valve replacement for severe aortic valve disease has been most satisfactory in many cases. It has provided a means of preventing otherwise inevitable early death and of greatly alleviating disability.

### REFERENCES

Brodeur, M. T. H., Sutherland, D. W., Koler, R. D., Starr, A., Kimsey, J. A., and Griswold, H. E. (1965). Circulation, *32*, 570.

Scannell, J. G., and Austen, W. G. (1966) Circulation, *33*, Supplement No. 1, p. 131.

